# Validation of an LC-MS/MS Method to Quantify the New TRPC6 Inhibitor SH045 (Larixyl *N*-methylcarbamate) and Its Application in an Exploratory Pharmacokinetic Study in Mice

**DOI:** 10.3390/ph14030259

**Published:** 2021-03-13

**Authors:** Xiao-Ning Chai, Friedrich-Alexander Ludwig, Anne Müglitz, Michael Schaefer, Hai-Yan Yin, Peter Brust, Ralf Regenthal, Ute Krügel

**Affiliations:** 1 Rudolf Boehm Institute for Pharmacology and Toxicology, Leipzig University, 04107 Leipzig, Germany; chaixn.1994@gmail.com (X.-N.C.); anne.mueglitz@medizin.uni-leipzig.de (A.M.); michael.schaefer@medizin.uni-leipzig.de (M.S.); 2 Acupuncture and Tuina School, Chengdu University of Traditional, Chinese Medicine, Chengdu 610075, China; yinhaiyan@cdutcm.edu.cn; 3 Department of Neuroradiopharmaceuticals, Institute of Radiopharmaceutical Cancer Research, Helmholtz-Zentrum Dresden-Rossendorf, 04318 Leipzig, Germany; f.ludwig@hzdr.de (F.-A.L.); peterbrustdeu@aol.com (P.B.); 4 Clinical Pharmacology, Rudolf Boehm Institute for Pharmacology and Toxicology, Leipzig University, 04107 Leipzig, Germany; ralf.regenthal@medizin.uni-leipzig.de

**Keywords:** channelopathies, larixol, labdane, LC-MS/MS, mice, pharmacokinetics, SH045, TRP channels, TRPC6 inhibitor

## Abstract

TRPC6 (transient receptor potential cation channels; canonical subfamily C, member 6) is widespread localized in mammalian tissues like kidney and lung and associated with progressive proteinuria and pathophysiological pulmonary alterations, e.g., reperfusion edema or lung fibrosis. However, the understanding of TRPC6 channelopathies is still at the beginning stages. Recently, by chemical diversification of (+)-larixol originating from *Larix decidua* resin traditionally used for inhalation, its methylcarbamate congener, named SH045, was obtained and identified in functional assays as a highly potent, subtype-selective inhibitor of TRPC6. To pave the way for use of SH045 in animal disease models, this study aimed at developing a capable bioanalytical method and to provide exploratory pharmacokinetic data for this promising derivative. According to international guidelines, a robust and selective LC-MS/MS method based on MRM detection in positive ion mode was established and validated for quantification of SH045 in mice plasma, whereby linearity and accuracy were demonstrated for the range of 2–1600 ng/mL. Applying this method, the plasma concentration time course of SH045 following single intraperitoneal administration (20 mg/kg body weight) revealed a short half-life of 1.3 h. However, the pharmacological profile of SH045 is promising, as five hours after administration, plasma levels still remained sufficiently higher than published low nanomolar IC_50_ values. Summarizing, the LC-MS/MS method and exploratory pharmacokinetic data provide essential prerequisites for experimental pharmacological TRPC6 modulation and translational treatment of TRPC6 channelopathies.

## 1. Introduction

The increasing knowledge about involvement of transient receptor potential cation channels (TRP) in a wide range of cell functions expedites the search for small molecular modulators useful to investigate their importance for physiological and pathophysiological processes in vivo. Among them, TRPC6 (canonical subfamily C, member 6) attracted interest as receptor-operated non-selective cation channel with considerable Ca^2+^ permeability, thereby mediating both increases in cytosolic Ca^2+^ concentration and membrane depolarization [1]. The channel activation is mediated by diacylglycerol (DAG), a messenger downstream of G_q/11_ protein-coupled receptors or of receptor tyrosine kinases that signal through phospholipases C (PLC) [2,3].

TRPC6 is localized in various cell types and tissues, e.g., kidney, heart, lung and various areas of the central nervous system [4]. In the kidney, TRPC6 is expressed throughout the glomeruli comprising cortex, the inner and outer medulla and podocytes, whose dysfunction impairs kidney filter function and causes edema and proteinuria as hallmarks of many and mostly progressive kidney diseases [5,6]. Non-genetic forms of such diseases like focal segmental glomerulosclerosis (FSGS), diabetic nephropathy, immune-mediated kidney diseases and renal fibrosis are attributed to TRPC6 dysregulation. However, gene mutations of the TRPC6, either as gain-of-function or loss-of-function, may also cause glomerulosclerosis and nephrotic syndrome [7].

In the lung, TRPC6 is highly expressed in vascular endothelia, precapillary pulmonary arterial smooth-muscle cells (PASMC), neutrophils and myofibroblasts, where it is associated with numerous pathophysiological pulmonary alterations, such as hypoxic vasoconstriction, pulmonary hypertension, lung ischemia-reperfusion edema (LIRE, a main reason for lung transplant failure and lung embolism), or lung fibrosis [8,9]. 

By an academic high-throughput screening in a chemically diverse library, Urban et al. proved that targeting of TRPC6 is feasible and identified several chemical entities with potency to block TRPC6, but all with only limited isotype-specificity to its closest relatives TRPC3/7 [10]. Considering that balsams, essential oils, or incense materials traditionally used for inhalation may contain biologic activities, a natural compound strategy to identify new TRPC6-blocking chemical entities in several plant extracts was embarked. A strong inhibitory activity on TRPC6 was found in conifer balsams associated with nonvolatile resins, but not with essential oils. The labdane diterpene (+)-larixol [11] isolated and crystallized from *Larix decidua* turpentine and, in particular, its congener larix-6-yl monoacetate displayed marked inhibitory potency and promising selectivity for the TRPC6-subtype associated with biological action [12]. 

Motivated by the renaissance in use of natural products in drug discovery spurred by Biology-Oriented Synthesis (BIOS) [13], these authors performed a systematic chemical diversification of the (+)-larixol scaffold by synthesis of several new congeners, followed by structure–activity relationship analyses and presented larixyl *N*-methylcarbamate, named SH045, as a compound with nanomolar affinity and 13-fold subtype selectivity over TRPC3 [14]. Furthermore, SH045 inhibited the 1-oleyl-2-acetyl-*sn*-glycerol (OAG)-induced Ca^2+^ entry through TRPC3/6 channels of rat PASMC and reduced edema formation after reperfusion of ischemic lungs explanted from mice, an ex vivo model of LIRE. 

To pave the way for use of these promising pharmacodynamic features of SH045, we provide here in a first step a robust validated liquid chromatography-tandem mass spectrometry (LC-MS/MS) method to quantify SH045 in blood plasma, and in a second step, a proof-of-concept pharmacokinetics trial in mice to enable the transition of SH045 as an experimental tool into preclinical disease models.

## 2. Results

### 2.1. Optimization of LC-MS/MS Conditions for SH045 and Internal Standard (IS)

To enable its application in preclinical in vivo studies, a sensitive method to quantify the new TRPC6 inhibitor SH045 in plasma was developed, covering optimization of high performance liquid chromatographic (HPLC) and tandem mass spectrometric (MS/MS) conditions as well as implementation of a suitable internal standard (IS). 

Generally, due to the chemical structure of SH045 containing a hydroxyl group and carbamate functionality, the analyte was expected to get sufficiently ionized during electrospray ionization (ESI) in positive mode, further enhanced by formic acid (0.1%) which was superior to ammonium formate (2 mM) as an additive in the chromatographic solvent system. 

Further, the chemical structure of the analyte also suggested that reversed-phase conditions are highly suitable for its chromatography. Hence, four different columns (A–D) were preselected from the wide spectrum of reversed-phase stationary phases available. In detail, the columns tested for gradient elution using acetonitrile with 0.1% formic acid were as follows: Column A—Luna C18(2) 100 Å (100 × 3.0 mm, 3 µm, Phenomenex, Aschaffenburg, Germany), column B —Luna C18 100 Å (50 × 2.1 mm, 5 µm, Phenomenex), column C—Zorbax Eclipse XDB-CN 80 Å (150 × 3.0 mm, 3.5 µm, Agilent Technologies, Waldbronn, Germany) and column D—Zorbax Eclipse XDB-C8 80 Å (100 × 3.0 mm, 3.5 µm, Agilent Technologies) (Supplementary Figure S1 and Table S1). Of them, both Luna C18 columns (A and B) are known to cause retention of analytes mainly by hydrophobic interactions, whereas polar and ionic interactions are more pronounced characteristics of phases with shorter alkyl chains, such as the Zorbax Eclipse XDB-C8 column (D). Besides, another column with “extra dense bonding”, the Zorbax Eclipse XDB-CN column (C), was tested to check whether its further increased propensity to polar interactions is of advantage. Furthermore, to achieve run times not longer than 20 min, appropriate column dimensions, namely in terms of length, inner diameter and particle size, were chosen as stated. To enable measurements with an even shorter run time and therefore higher throughput, column B, having a length of 50 mm and an inner diameter of 2.1 mm, was also included in the survey. 

The results of the comparison, which led to the decision to use the Zorbax Eclipse XDB-C8 column (D) for our study, are summarized in Supplementary Table S1, while the related multiple reaction monitoring (MRM) chromatograms are shown in Supplementary Figure S1. In brief, using columns A to D, gradient elution and injection of equal amounts of SH045, dissolved in pure solvent, the highest number of theoretical plates (*N*) was calculated for the XBD-C8 column (D), which therefore exhibited the highest column efficiency. In contrast, the Luna C18 column (B, 50 × 2.1 mm) showed the highest value in terms of peak area, nearly twice as much as observed for column D. However, this superiority diminished almost completely when standard samples of SH045 prepared from mouse plasma were measured, which was of relevance for the planned pharmacokinetic study. 

Asymmetry and tailing factors were calculated (Supplementary Table S1). Most ideal for plasma samples appeared to be the tailing factor for the Luna C18 column (B). However, the chromatogram clearly showed an unfavorable peak tailing (Supplementary Figure S1b) and therefore was not considered for further use. For the XBD-C8 column (D), a sufficient tailing factor was obtained consistent with an appropriate peak profile.

Taken together, among the columns tested, the Zorbax Eclipse XDB-C8 (column D) was revealed as the most suitable one and was finally chosen for the study. Using an optimized gradient method, SH045 eluted at 9.72 min during a total run time of 20 min. 

Since no isotopically labelled derivative of SH045 was available to serve as IS, the suitability of three possible well-described drug candidates, namely dexamethasone, imipramine and primidone [15], were examined together with the parent compound (+)-larixol. 

In brief, to enable MS detection for quick examination, their enhanced product ion (EPI) spectra were recorded. Based on the most intense signals of fragment ions, MRM methods were set up. Using them, the four potential IS were found to eluate at 4.64, 4.64, 1.96 and 9.46 min respectively, under the aforementioned optimized LC conditions for SH045. From this series, (+)-larixol (9.46 min) was preselected because of its retention time close to that of SH045 (9.72 min), while maintaining peak separation during the applied chromatographic method and so avoiding possible cross-talk during MS detection. 

**Figure 1:**
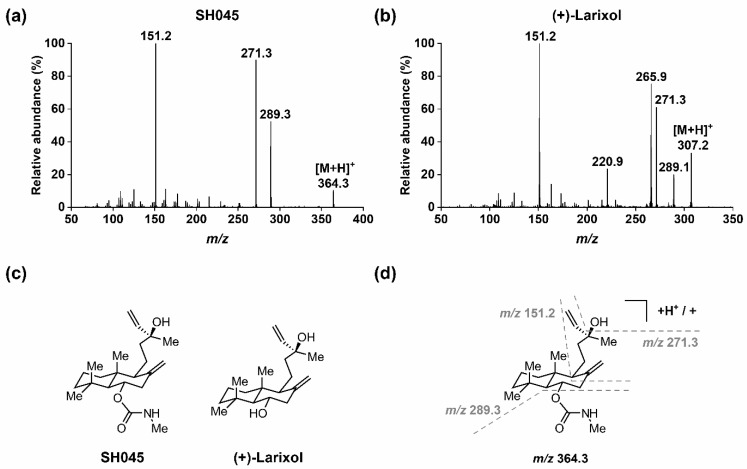
MS/MS of SH045 and (+)-larixol: (**a**) EPI spectrum of SH045 (product of *m*/*z* 364.3), (**b**) EPI spectrum of (+)-larixol (product of *m*/*z* 307.2), (**c**) chemical structures of SH045 and (+)-larixol and (**d**) explanation for fragmentation pattern in EPI spectrum of SH045.

Furthermore, (+)-larixol and its congener SH045, both belonging to the class of labdane diterpenes, were anticipated to show similar behavior during sample preparation due to their structural similarity. Hence, having confirmed that (+)-larixol had no influence on the extraction of SH045 from plasma nor it was detectable in plasma after administration of SH045, it was finally chosen as IS for method development and application (Figure 1). 

In order to quantify SH045 with the aid of (+)-larixol as IS, MS detection methods for both compounds were optimized using MRM in positive ion mode with ESI. In brief, solutions of the analytes (100 ng/mL) in water/acetonitrile (ACN) 1:1 (*v*/*v*) containing 0.1% formic acid were infused by a syringe pump, while an automated sample optimization procedure was performed. For SH045 and IS, the most intense MRM transitions were *m*/*z* 364.3→ *m*/*z* 151.2, *m*/*z* 364.3 → *m*/*z* 271.3 and *m*/*z* 364.3 → *m*/*z* 289.3, as well as *m*/*z* 307.2 → *m*/*z* 271.2, *m*/*z* 307.2 → *m*/*z* 220.9, *m*/*z* 307.2 → *m*/*z* 265.9 and *m*/*z* 307.2 → *m*/*z* 151.2, respectively. Of these, the latter showed highest signal intensities and were selected for detection. When recording the EPI spectrum for SH045 (Figure 1a), the product ion of *m*/*z* 289.3 was not the most intensive, however, it was used for quantitation since it provided the most intensive signal for detection by MRM. The optimized MRM conditions applied for detection of SH045 and IS throughout the study are specified in Table 1. 

In addition, a rationale for the fragmentation pattern observed in the EPI spectrum of SH045 (Figure 1a) is given in Figure 1d. Starting from the parent ion [M+H]^+^ at *m*/*z* 364.3, the *N*-methylcarbamate moiety was cleaved (-*m*/*z* 75), followed by an elimination of water (-*m*/*z* 18), which in turn resulted from the alkyl hydroxyl group in the molecule. Consequently, the latter was observed for (+)-larixol as well (Figure 1b), and the formation of a fragment ion at *m*/*z* 271.3 was similarly detected. Further, cleavage of the bicyclic ring system finally resulted in the main fragment ion of *m*/*z* 151.2 for both SH045 and (+)-larixol.

### 2.2. Plasma Sample Preparation

Based on the known very high sensitivity and selectivity of MS/MS detection methods and comprehensive successful experiences with protein denaturation techniques, we preferred simple and fast plasma protein precipitation to more expensive sample preparation procedures like liquid extraction or solid-phase extraction. To avoid any possible interferences due to different solvents with the water/ACN-based mobile phase, we focused on protein precipitation by ACN followed by centrifugation and filtration. We examined the MRM signal peak area of SH045 in the filtrate after precipitation without and with 0.1% formic acid in a ratio of plasma to solvent of 1:5, whereby the latter was less effective. Enhancing the ratio of plasma to pure ice-cold ACN from 1:5 to 1:10 without formic acid increased the peak area about two-fold. Method validation (see below) confirmed that the chosen technique provides complete extraction and meets all quality requirements for bioanalytical methods. 

### 2.3. Method Validation

The LC-MS/MS method was validated in accordance with the guidelines on bioanalytical method validation from the European Medicines Agency (EMA, 2011) and the U.S. Food and Drug Administration (FDA, 2018) [16,17], including selectivity, carry-over, linearity, limit of detection (LOD) and lower limit of quantification (LLOQ), accuracy and precision, matrix effect, recovery, stability and dilution integrity, which were critically evaluated.

#### 2.3.1. Selectivity, LOD, LLOQ, Carry-Over and Linearity

**Figure 2:**
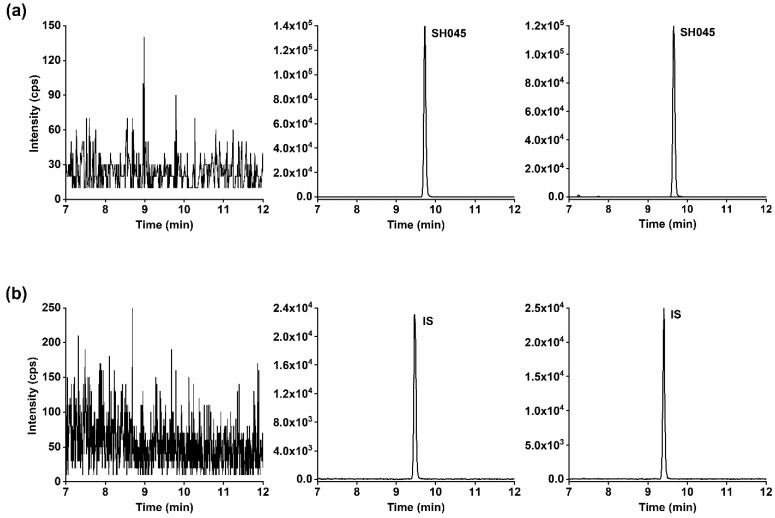
Representative MRM chromatograms of (**a**) SH045 and (**b**) (+)-larixol as IS in mouse blank plasma (**left panel**), blank plasma spiked with 100 ng/mL of SH045 and 5 µg/mL IS (**middle panel**) and in a mouse plasma sample obtained 3 h following administration of SH045 (20 mg/kg, i.p., **right panel**).

The chromatograms of SH045 and IS in spiked plasma and plasma after intraperitoneal (i.p.) administration of SH045 compared to blank plasma did not reveal any interferences (Figure 2). Furthermore, quantification of the percentage of blank plasma response at the retention times for SH045 and IS, compared to their intensity in the LLOQ sample, verified a high selectivity of the LC-MS/MS method. The blank plasma sample intensities amounted to 2.194% ± 0.359% of SH045 at LLOQ (2 ng/mL) and 0.036% ± 0.004% of IS at its present concentration (5 µg/mL, *n* = 6). 

Based on signal-to-noise ratios, LOD and LLOQ concentrations were determined to be 0.5 and 2.0 ng/mL, respectively. The LLOQ of 2.0 ng/mL also refers to the method sensitivity.

No carry-over effects were detected when blank plasma samples were analyzed following samples of the upper limit of quantification (ULOQ, 1600 ng/mL). Intensities of blank plasma samples of 4.3% for SH045 and 0.7% for IS were below the required limits of 20% and 5% of the LLOQ samples.

The linearity of the method was demonstrated by calibration curves with eight equally processed calibration samples in the range of 2 to 1600 ng/mL with excellent linear regression coefficients of *r*^2^ ≥ 0.998. The resulting average equation for SH045 quantification in plasma was *y* = *2.950 x* + *0.007*. The calibration performance was proven for each daily fresh prepared calibration samples automatically with a typical deviation (*c_calc_*/*c_actual_ x 100*) of less than 10%. Supplementary Table S2 exemplarily presents the calibration validation for SH045 for the inter-day accuracy of three subsequent days.

#### 2.3.2. Accuracy and Precision

Data of intra- and inter-day accuracy and precision assessed with spiked plasma at LLOQ and in quality control (QC) samples (L: low; M: medium; H: high) are summarized in Table 2. The intra- and inter-day accuracy values differed from −9.0% to 5.3% of nominal values, and the precision values were <9% for all concentration levels, confirming that the method met the guideline’s requirements to quantify SH045 in mouse plasma. 

#### 2.3.3. Extraction Recovery and Matrix Effect

To ensure the quality of sample preparation, the extraction recovery and matrix effects were tested at LLOQ and three QC sample levels. The values are presented in Table 3. The extraction recovery for SH045 ranged from 96.0% to 111.8% and for IS, a mean of 102.4% was determined. All relative standard deviations (RSD) were less than 8%. Concerning the matrix effects, no mean values differed more than 8% from nominal values and the RSD was below 5%, except 12.9% for LLOQ. Therefore, a sufficient recovery for both compounds was accepted and matrix effects could be excluded. 

#### 2.3.4. Stability

Good storage stability of both compounds qualifies the method for routine use. Stock solutions of SH045 and IS were stable, with accepted accuracy after freezing at −20 °C for 28 days. The working solutions of both compounds were stable as well when kept at 4 °C for 12 h. Further, approved accuracy of QC samples at three concentration levels of SH045 in plasma under different storage conditions, including three cycles of freezing and thawing, evidences its high stability. Results are shown in Table 4.

#### 2.3.5. Dilution Integrity

Examination of dilution integrity ensured that plasma samples out of the measurable range of an analyte could successfully be diluted to determine the original concentrations. Analysis of plasma spiked with SH045 (1800 ng/mL) and diluted two- and four-fold with blank plasma provided the required accuracy (Table 5).

### 2.4. Plasma Protein Binding (PPB)

The protein binding of SH045 in mouse plasma determined in vitro after ultrafiltration was very high. PPB determined at concentrations of 100 and 800 ng/mL SH045 in plasma was 97.8% ± 0.1% and 99.7% ± 0.1%.

### 2.5. Concentration-Time Profile of SH045 in Plasma

**Figure 3:**
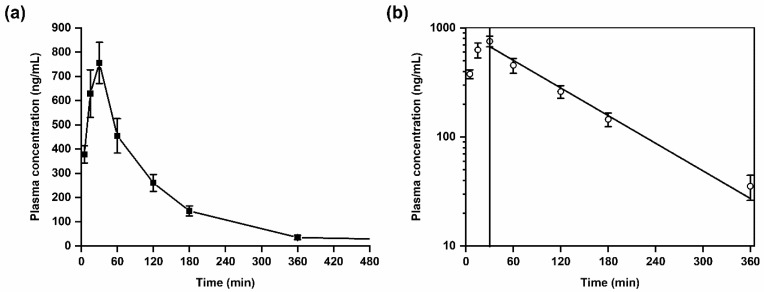
Plasma kinetics of SH045 in mice after intraperitoneal (i.p.) administration (20 mg/kg body weight). (**a**) Mean plasma concentration-time course of SH045, (**b**) semilogarithmic plot indicative for first-order elimination kinetics (Pearson’s r = −0.991).

The validated LC-MS/MS method was successfully applied in a first exploratory kinetic study in mice after administration of a single dose of SH045 (20 mg/kg body weight, i.p., *n* = 6) (Figure 3). Further, data received were used to calculate pharmacokinetic parameters based on a non-compartmental analysis (Table 6). 

SH045 was rapidly absorbed into the vascular system with a maximum plasma concentration (C_max_) of 756 ± 85 ng/mL at the time to maximum concentration (T_max_) of 30 min. The area under the plasma concentration-time curve for 6 h (AUC_0–6_) was calculated with 1410 ng/mL × h, whereas AUC from time zero to infinity (AUC_0_–_∞_) was 1473 ng/mL × h. This reflects an extrapolated AUC from the last measured time point to infinity, AUC_extra_, of only 4.3%. This low amount corresponds to a nearly complete systemic drug exposure covered by the non-compartment modeling and suggestive for adequate computing of the elimination function. 

The calculated half-life, T_1/2_, of SH045 was 1.3 h, however the mean residence time (MRT) was slightly longer than the T_1/2_. 

A semilogarithmic plot of mean drug concentrations in plasma versus time indicates that the drug elimination follows a first-order kinetics (Figure 3). The volume of distribution at steady state (V_ss_) of 25 L is very similar to the volume of distribution during the terminal phase (V_z_) of 25.5 L, which indicates a preferential extravascular appearance of SH045 (Table 6). 

## 3. Discussion 

A selective, accurate and reproducible bioanalytical method based on LC-MS/MS was developed and validated according to international guidelines to determine the new high-affinity and subtype-specific (+)-larixol congener, the TRPC6 inhibitor SH045, in rodent plasma. With this method, an exploratory pharmacokinetic profile of SH045 was obtained as a prerequisite for its use in preclinical experimental research, preferably focused on lung disease (LIRE, lung fibrosis) [18] and non-genetic forms of proteinuric kidney disease (FSGS) [7,19] animal models. As participation of TRPC6 is also discussed in neuropathic pain [20] and brain inflammation [21], the inhibitor may find further application in in vivo preclinical and clinical experimental therapies. 

The determination of preclinical active compounds like SH045 in plasma is a special challenge for method sensitivity due to restricted low blood volumes available from small rodents. The presented LC-MS/MS method is suitable for analysis of SH045 in 20 µL mice plasma or even less. This is of particular importance when blood is repeatedly collected from the same animal.

A crucial determinant for accuracy and precision in quantification is the adequate choice of an internal standard. The structurally related (+)-larixol is sufficiently stable and causes no interferences during sample preparation and detection by LC-MS/MS, where it behaves quite similar to SH045. Beyond that, these findings suggest that the method is adaptable to further (+)-larixol congeners.

In striving to understand channelopathies, several small molecular probes, synthetic and natural TRP family modulating compounds, were identified previously [1,22]. Although natural product-derived compounds often exhibit exquisite selectivity, specificity and potency [23], only a few natural compounds were shown to modulate TRPC subfamily function so far. Among them are the *Panax* saponin metabolite 20-*O*-β-D-glucopyranosyl-20(*S*)-protopanaxadiol, the monoterpene camphor from *Cinnamomum camphora* and hyperforin, the active compound of *Hypericum perforatum* [24,25], all of low potency and lack of TRPC6 subtype-selectivity. The (+)-larixol congener SH045 provided by Häfner et al. displays promising selectivity data for TRPC6 over TRPC3 and TRPC7 [14]. In addition, the congener exhibits a potent nanomolar half-maximal inhibitory concentration of IC_50_ = 5.8 nM to block inward currents in patch-clamp recordings and an IC_50_ = 63 nM to inhibit Ca^2+^ influx in stably TRPC6-transfected HEK293 cells [14]. 

Although the pharmacokinetic data reveal a rather short half-life of SH045, the plasma concentration is at least two-fold higher than the IC_50_ for Ca^2+^ influx at five hours after administration of 20 mg/kg, and therefore, is suggested to be still pharmacologically effective. 

Non-compartment analysis indicates that SH045 has a preferential extravascular distribution. Therefore, it is likely that SH045 achieves effective concentrations in target organs like lung and kidney. The drug elimination follows a first-order kinetics and allows further dose-effect titration or fine-tuning of future concentration-controlled drug-effect studies.

The high PPB found here may suggest a prolonged release of SH045 not only from plasma proteins but also from tissue proteins after distribution from the vascular system and might have considerable effects on biological responses to SH045. 

Given that repeated administration is required for treatment in experimental and clinical approaches, different kinetic features are conceivable when a high tissue binding results in a deep compartment, possibly leading to slower systemic elimination.

The i.p. route of drug administration is common practice in in vivo studies using rodent disease models. This route is easy to master, quick, reproducible, suitable for repeated treatments and, importantly, with low impact of stress. This agrees with efforts in refinements in animal use, especially in early animal studies and proof-of-concept studies where effects of target involvement rather than drug properties like formulations or clinical translation of pharmacokinetic parameters are the aim of investigation [18]. 

## 4. Materials and Methods

### 4.1. Materials

Larixyl-6-*N*-methylcarbamate ((1*S*,4*S*,4a*R*,8a*S*)-4-((*S*)-3-Hydroxy-3-methylpent-4-en-1-yl)-4a,8,8-trimethyl-3-methylenedecahydronaphthalen-1-yl methylcarbamate, SH045, TRPC6 inhibitor) was synthesized as described by Häfner et al. [14]. (+)-Larixol (internal standard, IS) was isolated from *Larix decidua* turpentine [14]. Kolliphor^®^ EL was obtained by Caelo (Hilden, Germany). Acetonitrile (ACN), dimethyl sulphoxide (DMSO), water and formic acid (LC-MS grade each) were purchased from Fisher Scientific (Schwerte, Germany).

### 4.2. HPLC and Tandem Mass Spectrometric Method (LC-MS/MS)

Analyses were performed with an Agilent 1260 Infinity quaternary HPLC system (Agilent Technologies, Waldbronn, Germany) consisting of a G4225A degasser, G1312B binary pump, G1367E autosampler, G1330B thermostat, G1316A column oven and G4212B diode array detector, coupled to a tandem QTRAP 5500 hybrid linear ion-trap triple quadrupole mass spectrometer (AB SCIEX, Concord, ON, Canada). Data were acquired and processed using Analyst software (Version 1.7.1, AB SCIEX). Linear regressions and calculations were done using Multiquant software (Version 2.1.1, AB SCIEX).

#### 4.2.1. Chromatographic Conditions 

Chromatographic separation was performed on a Zorbax Eclipse XDB-C8 column, 100 × 3.0 mm, 3.5 μm 80 Å (Agilent Technologies), at a flow rate of 0.7 mL/min and a column temperature of 25 °C. The solvent system consisted of water containing 0.1% formic acid (eluent A) and water/ACN 10:90 (*v*/*v*) containing 0.1% formic acid (eluent B). The optimized linear gradient for separation was run as follows (% ACN): 0–13 min, 20–90%; 13–16 min, 90%; 16–20 min, 20%. To prevent the mass spectrometer from contamination by biological matrix, the eluate was re-routed to waste for 2 min at the beginning of each measurement. All freshly extracted samples were stored in the autosampler at 6 °C until injection of 10 µL.

#### 4.2.2. Mass Spectrometric Conditions

The mass spectrometer was operated in positive electrospray ionization (ESI+) mode. The temperature of the turbo spray was maintained at 550 °C and the ESI needle voltage at 5500 V. Curtain gas, ion source gas I and ion source gas II were set at 35, 60 and 50 (arbitrary units), respectively. Detection of SH045 and IS by MRM was proceeded using previously optimized parameters, as specified in Table 1. Calibration and quantification of SH045 based on the ratios of the peak areas determined for SH045 and IS (A_SH045_/A_IS_).

EPI spectra for SH045 and IS were recorded with a scan rate of 10,000 Da/s, collisionally activated dissociation (CAD): High, and a collision energy (CE, arbitrary units) of 10 and 13, respectively.

### 4.3. Preparation of Standard Stock Solutions, Calibration and QC Samples 

Analytic stock solutions of SH045 and of IS (1 mg/mL each) were prepared in ACN and stored at −20 °C. Working solutions of SH045 and of IS (50 µg/mL) were freshly prepared by dilution with water/ACN 1:1 (*v*/*v*). Calibration and QC samples were prepared by addition of 2 μL SH045 working solutions and 2 μL IS solution to 18 μL of blank plasma, further processed identically to the preparation of plasma (see below). Freshly prepared calibration samples for SH045 in plasma comprised eight concentration levels analyzed in duplicates (2, 5, 10, 100, 200, 800, 1200 and 1600 ng/mL). 

### 4.4. Method Validation

The method was validated in accordance with the guidelines on bioanalytical method validation from the European Medicines Agency (EMA, 2011) and the U.S. Food and Drug Administration (FDA, 2018) [16,17]. Selectivity, carry-over, linearity, limit of detection (LOD) and lower limit of quantification (LLOQ), accuracy and precision, matrix effect, recovery, stability and dilution integrity were verified.

#### 4.4.1. Selectivity, LOD, LLOQ, Carry Over and Linearity

The selectivity of the method for SH045 and IS was assessed by the intensity ratio evoked by blank plasma as potential interfering endogenous biological matrix to that of SH045 at LLOQ and IS in spiked plasma. LLOQ refers to the lowest concentration of SH045 for quantification, whose signal-to-noise ratio is of at least five with acceptable accuracy and precision according to EMA [16] and defines the sensitivity of the method [17]. Selectivity is given if the blank plasma intensity ratio is ≤20% of LLOQ for SH045 and ≤5% for the IS in six independent samples. LOD refers to the lowest quantity of SH045 that can be distinguished from the zero control, defined by three times signal-to-noise ratio.

Carry-over effects were proven by injection of blank plasma following a high-concentration sample, here at ULOQ. Carry-over in blank plasma samples should be less than 20% of LLOQ and 5% of IS. 

The calibration range for SH045 was based on presumed concentrations in mouse plasma. Calibration curves were evaluated from analysis of calibration samples at eight different concentrations. Calibration curves were generated by linear regression of the peak area ratios of SH045/IS to nominal SH045 concentration in plasma, requiring a correlation coefficient of *r*^2^ > 0.99. SH045 concentrations were quantified by interpolation of corresponding intensity ratios on the calibration curves. 

#### 4.4.2. Intra- and Inter-Day Accuracy and Precision

Intra- and inter-day accuracy and precision were assessed at four concentration levels in six replicates on one day and on three subsequent days: LLOQ, low QC (LQC, <three times of LLOQ), middle QC (MQC, 30–50% of calibration curve range) and high QC (HQC, >70% of highest calibrator sample), processed identically to calibration and plasma samples. Intra- and inter-day accuracy, the closeness to nominal value, is accepted within 15% for QC samples, with the exception of LLOQ, accepted within 20%. Precision, the closeness of repeated individual measures, expressed as the coefficient of variation (CV) of the same runs, should be ≤15% for QC samples and ≤20% for LLOQ. 

#### 4.4.3. Extraction Recovery and Matrix Effects

Extraction recovery and matrix effects were determined at the four QC levels in six independent samples. The extraction recovery is expressed as percentage of peak area of SH045-spiked plasma to that from extracted plasma spiked with SH045. The matrix effect is examined by the ratio of peak areas of SH045 in extracted plasma to that of SH045 in solvent. Recovery and matrix effects are acceptable within accuracy and precision ranges given above.

#### 4.4.4. Stability Experiments

The stability of SH045 and IS in stock solutions stored at −20 °C for 28 days and in working solutions stored at 4 °C for 12 h was determined comparing the analyzed with the nominal concentration and accepted when accuracy was within 15%. 

Further, stability of SH045 in plasma samples was determined at three QC levels under various conditions: short-term stability after thawing and storage at room temperature for 4 h, long-term stability after storage at −20 °C for one month, post-preparative stability at autosampler storage (6 °C) for 12 h after extraction and after three cycles of freezing at −20 °C for 24 h and complete thawing. The accuracy of the mean concentration at each of the three QC levels was accepted within ±15% of nominal concentration. All stability experiments were carried out in three replicates.

#### 4.4.5. Dilution Integrity

To confirm that dilution of plasma samples with high concentrations has no impact on the measured concentration, blank plasma was spiked with SH045 in a concentration above the ULOQ and diluted two- and four-fold with blank plasma. The accuracy should not vary more than ±15% and precision should be ≤15%.

### 4.5. Plasma Protein Binding (PPB)

PPB of SH045 in mice plasma was determined at two concentrations, 100 and 800 ng/mL, using ultrafiltration. For that, 2 µL of working solutions of SH045 in DMSO (final concentration 1%) were mixed with 298 µL blank plasma and incubated at 37 °C for 30 min. To determine the total concentration of SH045, 60 µL sample were taken out. The remaining 240 µL of spiked plasma were transferred to a centrifugal filter (Centrifugal Filter Units, 30 kDa cut-off, Millipore, County Cork, Ireland) and centrifuged at room temperature for 10 min at 1000× *g*. The filtrates were analyzed for the unbound SH045. The PPB was calculated as PPB (%) = (C_t_ − C_f_)/C_t_ × 100, with C_t_ as total SH045 concentration in spiked plasma and C_f_ as unbound SH045 concentration in the filtrate.

### 4.6. Animals, Drug Administration and Plasma Sample Preparation

The practicability of the validated method for SH045 quantification was verified in a first exploratory pharmacokinetic study in mice. Male C57BL/6 mice (25–30 g body weight, Charles River, Sulzfeld, Germany) were housed in groups under standard conditions, allowed access to lab chow and water ad libitum. Animal experiments were performed according to the national regulations of animal welfare and were ethically approved by the local committee on animal welfare and the local authorities. Animal experiments were performed under strict consideration of the 3R principles (replace, reduce, refine), fully complying with the ARRIVE guidelines [26]. 

Solutions of SH045 for i.p. administration were prepared with DMSO (final concentration 0.5%) and Kolliphor^®^ EL (5% in water). SH045 was administered i.p. as a single dose of 20 mg/kg body weight. Blood was collected from mice tail vein at 5, 15, 30, 60, 120, 180 and 360 min after administration via heparinized catheters. After centrifugation at 4000× *g* at 4 °C for 10 min, plasma was stored at −80 °C until analysis. 

For sample preparation, 20 µL of thawed plasma were mixed with 2 µL of IS (50 µg/mL), vortexed for 30 s, added with 178 µL ice-cold ACN and shaken vigorously for 5 min. After centrifugation at 104,000× *g* for 15 min, the supernatant was filtered through a 0.2 µm pore-size syringe filter (Phenex-RC 4 mm, Phenomenex, Aschaffenburg, Germany) and stored in the autosampler at 6 °C until analysis by LC-MS/MS, as mentioned above. 

### 4.7. Plasma Kinetics of SH045

The evaluation of basic pharmacokinetic parameters of SH045 (20 mg/kg, i.p.) following a single dose was performed by non-compartmental analysis using a commercially available software program Kinetica, version 5.1 (Thermo Fisher Scientific, Waltham, MA, USA). The C_max_ and T_max_ were obtained directly from the plasma concentration-time curve. The slope of the terminal phase of the SH045 concentration profile (ʎz) was estimated as absolute values from >4 data points using a log-linear regression analysis. 

The area under the plasma concentration-time curve (AUC) for the time (t) providing the final measurable concentration (AUC_last_) was calculated using the log-linear trapezoidal rule. 

The AUC_0–∞_ was then determined as AUC_last_ + C_last_/ʎz, where C_last_ is the last measurable concentration. The systemic total body clearance from plasma (CL_p_) was calculated as applied dose divided by AUC_0–∞_. MRT was calculated as AUMC_0–∞_/AUC_0–∞_, where area under the first-moment curve (AUMC) was defined as area under the curve of the product of time and the plasma drug concentration versus time from zero to infinity. 

The volume of distribution, V_Z_, was calculated as CL_p_/ʎz. Finally, the AUC_extra_ extrapolates the AUC from the last measured time point to infinity.

### 4.8. Calculations and Statistical Analysis

All data are presented as mean ± standard error of mean (SEM), except where otherwise specified. RSD refers to the relative standard deviation used to describe recovery and matrix effects. OriginPro 2017G (OriginLab, Northampton, MA, USA) was used for graphical representations. Other analytical software is indicated in the respective sections.

## 5. Conclusions

The validated method now enables to prove bioavailability of SH045 and is likely to be adapted to related compounds like other carbamate or acetate derivatives, which could be of relevance too. Finally, the developed LC-MS/MS method provides the basis for determination of crucial tissue concentration profiles of SH045 to verify its effectiveness in the target organs. 

Pharmacokinetic data achieved with the presented LC-MS/MS method allow the use of the new TRPC6 inhibitor SH045 in preclinical approaches and may inspire future studies to explore physiological and pathophysiological functions of this member of the TRP channel family.

## Figures and Tables

**Table 1:** Optimized conditions for detection of SH045 and IS by MRM in positive ESI mode (* arbitrary units).

Analyte	Parent ion (*m*/*z*)	Production (*m*/*z*)	Collision Energy *	Collision Gas	Entrance Potential *	De-Clustering Potential *	Collision Cell Exit Potential *	Scan Time (ms)
SH045	364.3	289.3	9	Medium	10	50	14	100
IS	307.2	151.2	21	Medium	10	66	10	100

**Table 2:** Intra- and inter-day accuracy and precision for SH045 detection in mice plasma (*n* = 6).

QC Level	Nominal Conc. (ng/mL)	Intra-Day			Inter-Day		
		Mean ± SEM (ng/mL)	Accuracy (%)	Precision (%)	Mean ± SEM (ng/mL)	Accuracy (%)	Precision (%)
LLOQ	2	1.8 ± 0.1	91.0	5.2	1.9 ± 0.1	95.4	4.6
LQC	5	5.1 ± 0.1	101.6	7.6	5.2 ± 0.2	104.2	8.3
MQC	800	801 ± 12	100.2	6.6	796 ± 23	99.4	7.2
HQC	1200	1264 ± 13	105.3	4.3	1264 ± 26	105.3	5.1

**Table 3:** Extraction recovery and matrix effect of SH045 in mice plasma (*n* = 6).

QC Level	Nominal Concentration (ng/mL)	Recovery (%)		Matrix Effect (%)	
		Mean (%)	RSD (%)	Mean (%)	RSD (%)
LLOQ	2	111.8	4.4	91.6	12.9
LQC	5	109.1	7.3	102.1	4.1
MQC	800	96.0	3.8	96.9	3.6
HQC	1200	96.0	3.6	97.0	3.7
IS	50,000	102.4	5.6	103.8	4.1

**Table 4:** The stability of SH045 in working solutions and plasma samples (*n* = 3)**.**

Assessment		Conditions	Nominal Concen-Tration (ng/mL)	Mean ± SEM (ng/mL)	Accuracy (%)
Stock solution	SH045	−20 °C, 28 days	1000	1066 ± 9	106.6
	IS		50,000	49,610 ± 419	99.2
Working solution	SH045	4 °C, 12 h	1000	1037 ± 10	103.7
	IS		50,000	49,818 ± 944	99.6
QC samples	Short-term stability	25 °C, 4 h	5	5.4 ± 0.1	107.6
			800	816 ± 2	101.9
			1200	1186 ± 36	98.8
	Stability in autosampler	6 °C, 12 h	5	5.0 ± 0.2	99.0
			800	807 ± 28	100.8
			1200	1256 ± 50	104.7
	Freeze and thaw stability	−20 °C, 12 h, 3 cycles	5	4.9 ± 0.2	97.2
			800	721 ± 8	90.1
			1200	1083 ± 41	90.2
	Long-term stability	−20 °C, 30 days	5	5.4 ± 0.3	107.3
			800	763 ± 9	95.4
			1200	1199 ± 29	99.9

**Table 5:** Dilution integrity of SH045 in mice plasma (*n* = 6).

Analyte	Concentration Spiked (ng/mL)	Dilution Fold	Mean ± SEM (ng/mL)	Accuracy (%)	Precision (%)
SH045	1800	1:2	854 ± 15	94.9	4.4
		1:4	396 ± 4	88.1	2.3

**Table 6:** Non-compartment analysis of pharmacokinetic parameters in plasma after single-dose administration of SH045 (20 mg/kg body weight, i.p.) in mice.

Parameter	T_1/2_ (h)	T_max_ (h)	C_max_ (ng/mL)	AUC_(0–6)_ (ng/mL × h)	AUC_0–∞_ (ng/mL × h)	AUC_extra_ (ng/mL × h)	% AUC	MRT (h)	V_ss_ (L)	Clearance (CL)_Plasma_ (mL/min)
Value	1.31	0.5	756	1410	1473	63.7	4.3	1.9	25	226

## Data Availability

The data presented in this study are available on request from the corresponding authors.

## Supplementary Materials

The following are available online at https://www.mdpi.com/1424-8247/14/3/259/s1, Figure S1: Peak shapes of SH045 in (a) pure solvent and in (b) plasma after separation at different columns, Table S1: Features of considered columns, LC method, peak shapes for SH045 in solvent and mice plasma, Table S2: Calibration validation for the inter-day accuracy for SH045 of three subsequent days.
