# Supplementary material for: Validation of an LC-MS/MS Method to Quantify the New TRPC6 Inhibitor SH045 (Larixyl N-methylcarbamate) and Its Application in an Exploratory Pharmacokinetic Study in Mice
Source: Pharmaceuticals (Basel). 2021 Mar 13;14(3):259. doi: 10.3390/ph14030259 (PMC8000919; doi:10.3390/ph14030259)
Supplement: Supplementary file 1 [file pharmaceuticals-14-00259-s001.pdf]

pharmaceuticals

*Supplementary material*

# Validation of an LC-MS/MS method to quantify the new TRPC6 inhibitor SH045 (larixyl *N*-methylcarbamate) and its application in an exploratory pharmacokinetic study in mice

**Xiao-Ning Chai [1,2†], Friedrich-Alexander Ludwig [3†], Anne Müglitz [1], Michael Schaefer [1], Hai-Yan Yin [2], Peter Brust [3], Ralf Regenthal [4††] and Ute Krügel [1††*]**

1 Rudolf Boehm Institute for Pharmacology and Toxicology, Leipzig University, 04107 Leipzig, Germany; chaixn.1994@gmail.com (X.N.C.); anne.mueglitz@medizin.uni-leipzig.de (A.M.); michael.schaefer@medizin.uni-leipzig.de (M.S.); ute.kruegel@medizin.uni-leipzig.de
2 Acupuncture and Tuina School, Chengdu University of Traditional, Chinese Medicine, 610075 Chengdu, China; yinhaiyan@cdutcm.edu.cn
3 Department of Neuroradiopharmaceuticals, Institute of Radiopharmaceutical Cancer Research, Helmholtz-Zentrum Dresden-Rossendorf, 04318 Leipzig, Germany; f.ludwig@hzdr.de; peterbrustdeu@aol.com
4 Clinical Pharmacology, Rudolf Boehm Institute for Pharmacology and Toxicology, Leipzig University, 04107, Leipzig, Germany; ralf.regenthal@medizin.uni-leipzig.de
* Correspondence: ute.kruegel@medizin.uni-leipzig.de
†, †† These authors contributed equally.

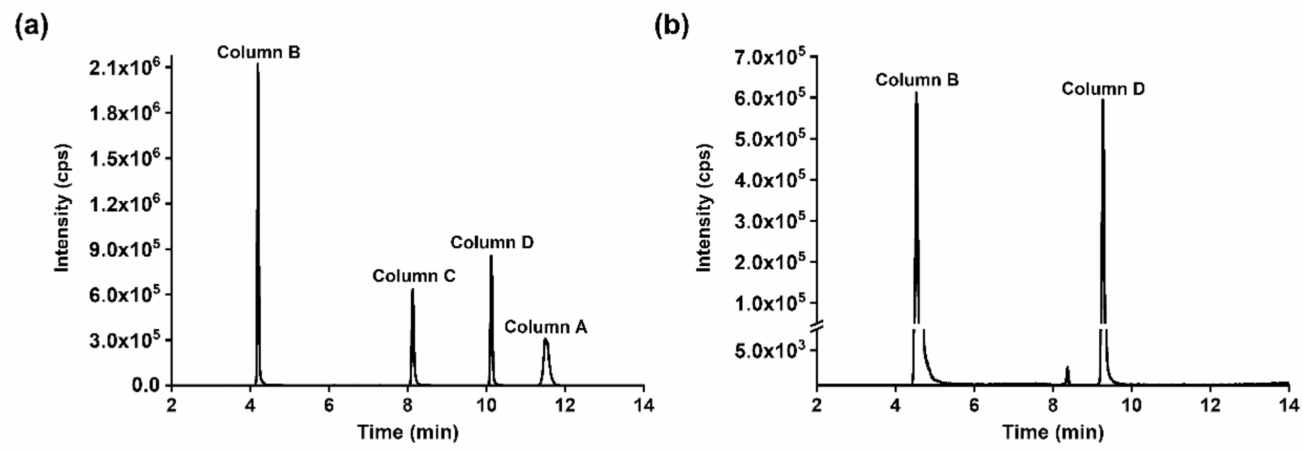


**Figure S1.** Peak shapes of SH045 in (a) pure solvent and in (b) plasma after separation at different columns: A - Luna C18(2) 100 Å (100 × 3.0 mm, 3 µm); B - Luna C18 100 Å (50 × 2.1 mm, 5 µm); C - Zorbax Eclipse XDB-CN 80 Å (150 × 3.0 mm, 3.5 µm); D - Zorbax Eclipse XDB-C8 80 Å (100 × 3.0 mm, 3.5 µm), see also **Table S1**.

**Table S1.** Features of considered columns, LC method, peak shapes for SH045 in solvent and mice plasma.

| | Solvent[a] | | | | Plasma[b] | |
|---|---|---|---|---|---|---|
| Column[c] | A | B | C | D | B | D |
| Size (mm) | 100 × 3.0 | 50 × 2.1 | 150 × 3.0 | 100 × 3.0 | see left | see left |
| Particle Size (μm) | 3 | 5 | 3.5 | 3.5 | | |
| Stationary phase | C18 | C18 | Cyano | C8 | | |
| Gradient (ACN %) | 0-13 min, 20-90%; 13-16 min, 90%; 16-20 min, 20% | 0-5 min, 40-90%; 5-6 min, 90%; 6-8 min, 40% | see A | see A | 0-5 min, 49-90%; 5-6 min, 90%; 6-8 min, 49% | see left |
| Injection volume (μL) | 0.4 | 0.4 | 0.4 | 0.4 | 10 | 10 |
| Flow rate (mL/min) | 0.7 | 0.4 | 0.7 | 0.7 | 0.4 | 0.7 |
| SH045 retention time (min) | 11.52 | 4.20 | 8.13 | 10.12 | 4.54 | 9.27 |
| Peak area (cps × min) | $3.17×10^6$ | $5.33×10^6$ | $2.58×10^6$ | $2.90×10^6$ | $2.51×10^6$ | $2.01×10^6$ |
| Height (cps) | $3.26×10^5$ | $2.21×10^6$ | $6.65×10^5$ | $8.36×10^5$ | $6.86×10^5$ | $6.14×10^5$ |
| FWHM (min) | 0.15 | 0.03 | 0.06 | 0.05 | 0.05 | 0.05 |
| Theoretical plates (*N*)[d] | 32,676 | 108,068 | 101,716 | 226,950 | 45,675 | 190,427 |
| Asymmetry | 1.308 | 1.017 | 1.200 | 1.422 | 0.985 | 1.216 |
| Tailing factor | 1.200 | 1.045 | 1.250 | 1.226 | 1.009 | 1.124 |

[a] 8.58 μg/mL SH045 in water/ ACN 1:1 (*v/v*). [b] 909 ng/mL SH045 in mice plasma. [c] column A: Luna C18(2) 100 Å (Phenomenex, Aschaffenburg, Germany), column B: Luna C18 100 Å (Phenomenex), column C: Zorbax Eclipse XDB-CN 80 Å (Agilent Technologies, Waldbronn, Germany), and column D: Zorbax Eclipse XDB-C8 80 Å (Agilent Technologies) [d] Theoretical plates was calculated as: $N = 5.54 \times (R.t./FWHM)^2$ where *R.t.* is the retention time of SH045 and FWHM is the Full Width at Half Maximum of peak height.

**Table S2.** Calibration validation for the inter-day accuracy for SH045 of three subsequent days.

| Calibration sample nominal concentration (ng/mL) | Concentrations found, mean ± SEM (ng/mL) | Accuracy (%) | Precision (%) |
|---|---|---|---|
| 2 | 2.0 ± 0.1 | 98.2 | 8.7 |
| 5 | 5.1 ± 0.2 | 101.8 | 5.3 |
| 10 | 9.8 ± 0.3 | 97.8 | 5.8 |
| 100 | 101± 5 | 100.6 | 7.7 |
| 200 | 193 ± 6 | 96.8 | 5.0 |
| 800 | 796 ± 1 | 99.5 | 0.2 |
| 1200 | 1228 ± 17 | 102.3 | 2.4 |
| 1600 | 1588 ± 53 | 99.3 | 6.1 |
